# Supplementary material for: Adaptive evolution in a conifer hybrid zone is driven by a mosaic of recently introgressed and background genetic variants
Source: Commun Biol. 2021 Feb 5;4:160. doi: 10.1038/s42003-020-01632-7 (PMC7864969; doi:10.1038/s42003-020-01632-7)

Supplementary information for:

Adaptive evolution in a conifer hybrid zone is driven by a mosaic of recently introgressed and background genetic variants

Mitra Menon[1*], Justin C. Bagley[2], Gerald F.M Page[3], Amy V. Whipple[4], Anna W. Schoettle[5], Christopher J. Still[3], Christian Wehenkel[6], Kristen M. Waring[7], Lluvia Flores-Renteria[8], Samuel A. Cushman[9] & Andrew J. Eckert[10]

Supplementary methods & results A: Assessing the sensitivity of various across individual cutoffs to detect adaptive introgression from *P. flexilis*

Since INTROGRESS[1] assesses ancestry enrichment on an individual SNP basis, we utilised a stringent multiple testing correction and a series of individual tree level cutoffs to determine SNPs that were exceptionally introgressed from *P. flexilis* background. Out of the 62,992 SNPs, 36,371 passed the Bonferroni corrected *p*-value threshold for displaying exceptional patterns of introgression. For each tree, we then retained only SNPs where the fitted estimate for *P. flexilis* ancestry was outside of the upper 95% confidence interval of *P. flexilis* ancestry null distribution. Using this two-step process, the distribution of significantly introgressed SNPs across individuals varied from 2853 (across all 950 trees) to 30,885 (in at-least one individual). We used a series of cutoffs ranging from significantly introgressed in at least 10% to 50% of individuals to classify a SNP as displaying signatures of adaptive introgression across the hybrid zone.

At 10% we identified a total of 29,560 outliers with *P. flexilis* ancestry enriched (see equation in methods section of main text) for beginning of frost free period (bFFP), frost free period (FFP), autumn degree days below zero ^{0}C (DD_0_at), winter precipitation as snow (PAS_wt), spring precipitation as snow (PAS_sp), spring degree days above 5^{0}C (DD5_sp), spring degree days below 18^{0}C (DD_18_sp), summer precipitation (PPT_sm), spring solar radiation (RAD_sp), autumn average temperature (Tavg_at) and spring Hargreaves climatic moisture deficit (CMD_sp) at a *p*-value cutoff of 0.01. From 20% - 50% cutoffs we identified 28,763 to 26,116 outliers, respectively. For these we noted a similar enrichment of *P. flexilis* ancestry for freezing temperature related variables (listed above) at *p*-value cutoffs of 0.01 and 0.001 using the permutation approach detailed in the main text.

Using a similar approach, we identified 24,138 SNPs exhibiting high ancestry from *P. strobiformis* at 20% individual based cutoff. Since contemporary gene flow was restricted between *P. flexilis* and the hybrid zone and the current study only focuses on recent introgression, we restrict this study to only identifying loci exhibiting excess retainment of *P. strobiformis* ancestry and don't attempt to infer their association with environmental gradients.

Supplementary methods & results B: Alternative approach to detect signatures of adaptive introgression based on climate similarity between the hybrid populations and *P. flexilis.*

Overall low levels of species differentiation can bias inference of adaptive introgression as it becomes difficult to distinguish ancestral segregating polymorphism due to incomplete lineage sorting from species-specific variants. Although the parametric approach within genomic cline analysis with a stringent *p*-value cutoff[2] is recommended under this situation to identify signatures of adaptive introgression, the identification of a large number of loci showing exceptional patterns of introgression might still be prone to false positives. To further test whether our inference of adaptive introgression was robust in the face of these biases, we utilised a simple correlation based approach that tested whether hybrid populations that matched the environment conditions of sampled of *P. flexilis* **(a)** were less divergent from *P. flexilis* in the median allele frequency difference than expected by chance (i.e., correlate AF_{diff} with Env_{diff}) and **(b)** had higher median proportion of loci with *P. flexilis* ancestry (i.e., correlate AF_{diff} with Anc_{PF}). The environment of *P. flexilis* was defined as the median value of a given environmental gradient across all the 12 sampled populations. This analysis was conducted for each environmental gradient by utilising the outlier SNPs identified through Bayenv (see main text for details). Further, we compared our observed correlation coefficient against a bootstrap distribution that was matched on two-dimensional bins of minor allele frequency and F_{ST}.

For approach **(a)** we expected a significant positive correlation between AF_{diff} with Env_{diff} for environmental gradients driving adaptive introgression from *P. flexilis.*
We noted a strong positive correlation (Pearson's $r = 0.8$ to 0.5) for gradients associated with radiation (RAD_sp), winter temperatures (NFFD_wt, Tmin_wt, MCMT, FFP), degree days (DD18_sp, DD5_sp, DD5_wt, DD_0) and continentality (TD). Using the bootstrap approach, most variables, except for those associated with precipitation, drought, radiation and summer snow, were significant ($p < 0.025$).

For approach **(b)** we expected a significant negative correlation between AF_{diff} with Anc_{PF}. We noted a strong negative correlation (Pearson's $r = -0.9$ to -0.7) for variables associated with continentality (TD), winter temperature (Tave_wt, MCMT) and degree days (DD_0_at, DD_18_wt). The lower tail of the bootstrap distribution ($p < 0.025$) was dominated by variables associated with degree days below zero (DD_0_wt, DD_0_at), growing degree days

(DD5_sp, DD5_wt, DD_18_wt), frost period (FFP), freezing temperatures (Tmin_sp, Tave_wt, EXT), continentality (TD) and reference evapotranspiration (Eref).

Supplementary methods & results C: Processing of raw ddRADseq data.

We utilised a series of publicly available pipelines and custom scripts to process the sequence files and obtain the starting set of SNPs used in this study. Processing of individual FASTQ files was conducted using dDocent v 1.0[3]. This process included the following steps implemented within dDocent a) read quality filtering with a PHRED score cutoff of 10, b) read grouping and a reference sequence assembly using the clustering algorithm CD-HIT with a within individual read coverage cutoff of 3 and an across individual read cutoff of 4, c) generating a reference assembly using the longest read in each cluster within a sequence similarity threshold of 0.89, d) read mapping to the *de novo* constructed reference assembly using BWA (default parameters except maximum mismatch value of 4 and a gap penalty of 6) and SNP calling using the default parameters in FREEBAYES v 0.9.10[4].

Downstream processing of the resulting variant call format (VCF) file was performed using VCFTOOLS v.0.1.153[5] to remove indels, retain biallelic SNPs with at least 50% data. We used a minor allele frequency (MAF) cutoff of 0.001 to retain rare variants likely reflecting signatures of selection and to avoid removing populations with small number of individuals that may contain unique variants. Further processing involved the use of an absolute F_{IS} cutoff of 0.5 (keeping SNPs with values between 0.5 and −0.5), a minimum PHRED quality score of 20 and maximum depth per read set as the 50% percentile of depth distribution. These steps were performed using custom python scripts and yielded a total of 72,889 SNPs, which were used as the starting dataset for all subsequent analyses.

Supplementary methods & results D: Parameter determination for LD network analysis

Most adaptive traits in natural populations are polygenic in nature and hence tend to co-vary in their allele frequencies across populations. To capture the polygenic signature of adaptive evolution within the *P. strobiformis-P. flexilis* hybrid zone, we conducted LDna[6] to identify distinct clusters of SNPs exhibiting strong associations amongst themselves. Within LDna, the stringency of outlier cluster (OC) cutoff depends on the constant (Φ) that scales the median absolute deviation across all λ values in the tree and the minimum number of edges (E_{min}) [6]. Using a hierarchical tree constructed using Φ and E_{min}, the change in median LD among SNPs within a cluster before and after merger is given by λ and OCs are identified by large λ values above the stringency cutoff. Thus, the change in LD when two clusters merge is measured by λ. To determine the appropriate value of E_{min} and Φ, we used a subset of the 100 pairwise LD matrices to generate a series of trees with values of E_{min} ranging from 4 to 10 but holding Φ constant at 2. Each LD matrix consisted of the 500 unique Bayenv outlier SNPs and 500 randomly generated putatively neutral SNPs that were matched in minor allele frequency bins. We were consistently able to recover similar OCs across varying values of E_{min} , hence we decided to utilize an E_{min} of 9. Our choice of parameters is justified given the large genome sizes of conifer species, low coverage obtained through ddRADseq and on average rapid decay of LD (*cf.* ref 7).

The LDna approach at an E_{min} of 9 and Φ of 2 identified a range of OCs (1 to 20) across the 100 replicate sets of SNPs. We only focused on OCs that did not contain any nested clusters (designated as SOC in ref. 6), as within a replicate run these would contain mutually exclusive sets of SNPs, such that each OC likely represents a different selective pressure.

Supplementary references

1. Gompert Z & Buerkle AC. *introgress: methods for analyzing introgression between divergent lineages*. R package version 1.2.3 (2012).
2. Janoušek V, *et al*. Genome-wide architecture of reproductive isolation in a naturally occurring hybrid zone between *Mus musculus musculus* and *M. m. domesticus*. *Mol. Ecol.* **21**: 3032-3047. (2012).
3. Puritz JB, Hollenbeck CM & Gold JR. dDocent : a RADseq, variant -calling pipeline designed for population genomics of non -model organisms. *PeerJ*, **2:** e431. (2014).
4. Garrison E, Marth G. Haplotype-based variant detection from short-read sequencing. *arXiv preprint arXiv:1207.3907 [q-bio.GN]*. (2012).
5. Danecek P, *et al*. The Variant Call Format and VCFtools, *Bioinformatics*. (2011).
6. Kemppainen P *et al.* Linkage disequilibrium network analysis (LDna) gives a global view of chromosomal inversions, local adaptation and geographic structure. *Mol. Ecol. Resour.* **15**: 1031–1045. (2015).
7. Kemppainen P, LDna: vignettes. Version 0.64. https://github.com/petrikemppainen/LDna/tree/master/vignettes

Supplementary figure 1: Basic summary statistics for three groups: *P. flexilis, P. strobiformis* and hybrid zone.

(a) Boxplot representing the distribution of expected and observed heterozygosity for each of the three groups. b) Boxplot representing the distribution of among population differentiation (F_{ST}) for each of the three groups. Each box represents the 1st and the 3rd quartile with the median given by the midline and the whiskers extending to 1.5 times the Inter-quantile range.

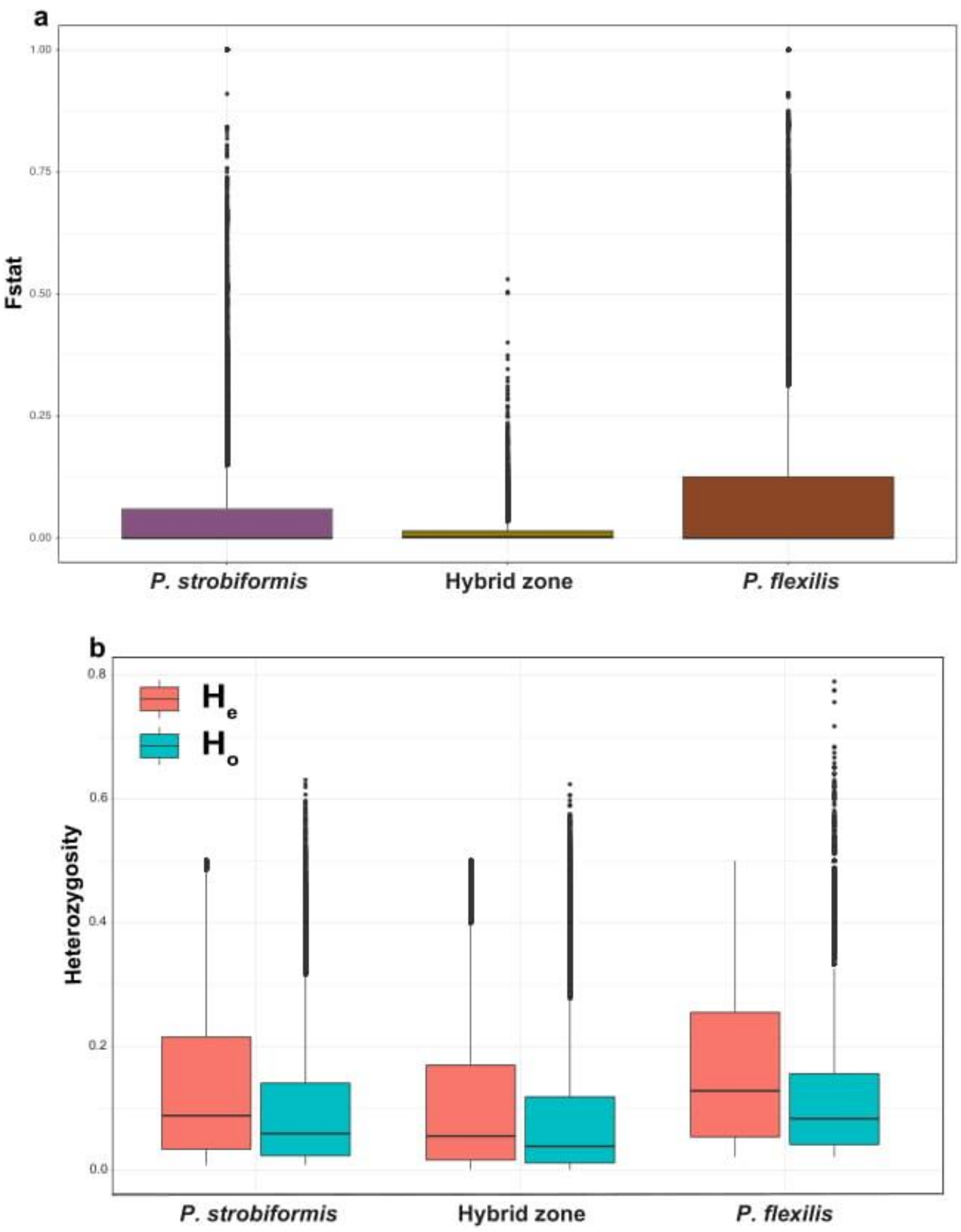

Supplementary figure 2: **STRUCTURE plots representing per individual tree's ancestry from *P. flexilis* and *P. strobiformis.***

(a) Ancestry proportions using only putatively neutral set of SNPs (i.e. SNPs that were not identified as Bayenv outliers) and (b) Ancestry proportions using only the 500 SNPs identified as outliers via Bayenv.

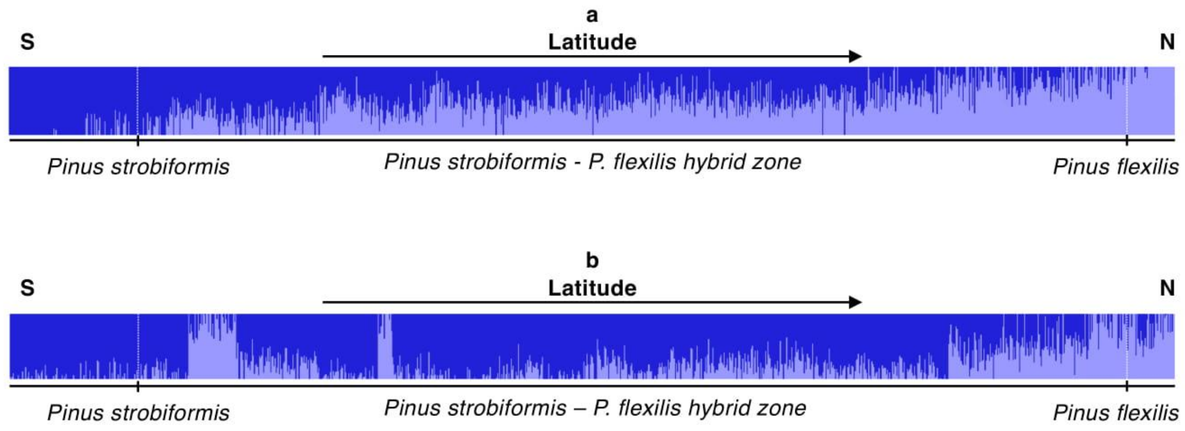

Supplementary figure 3: **Schematic representation of the sequential variance partitioning approach used in RDA to estimate pure, joint and confounded effects.**

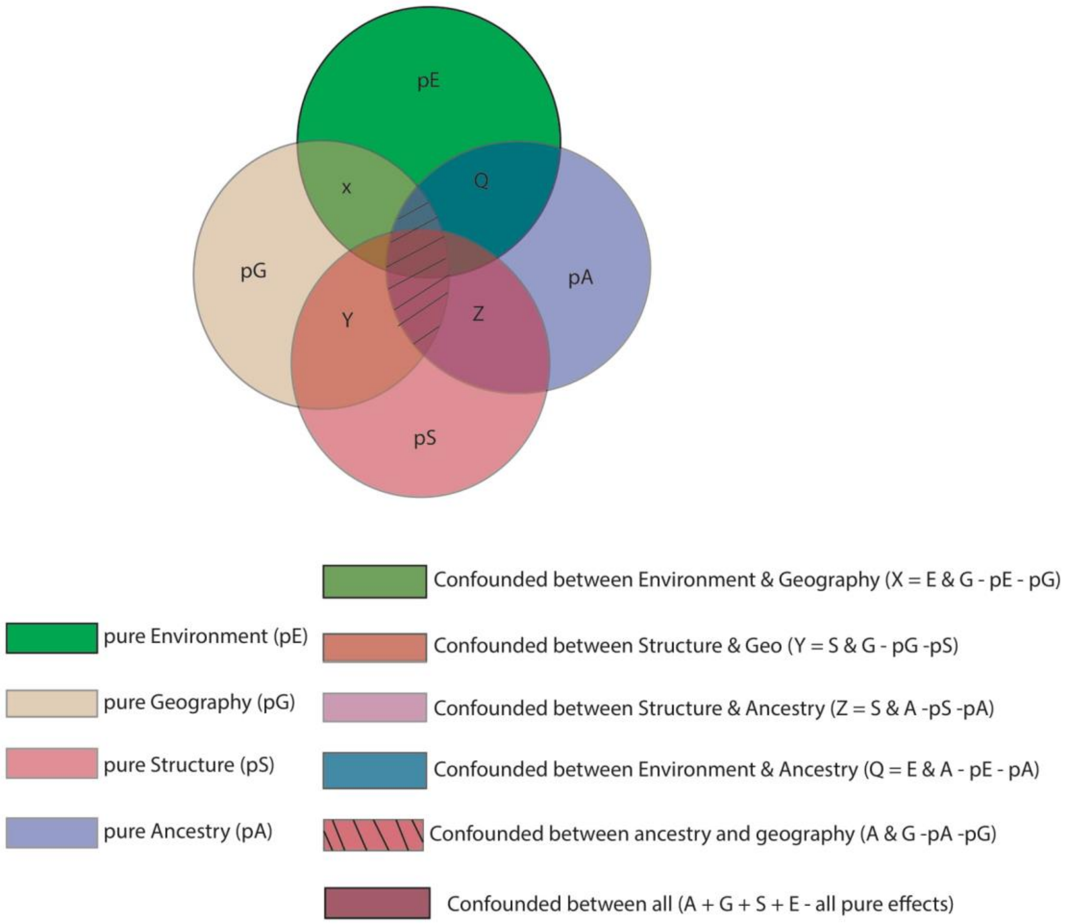

Supplementary figure 4: Evaluation of convergence and mixing across three Markov-chain runs during the variance-covariance estimation stage of Bayenv2.
(a) Trace plots of the determinant of the variance-covariance matrix for all 500,000 iterations across three independent Markov chains. (**b**) Trace plots after the burn-in, starting at iteration 20,000.

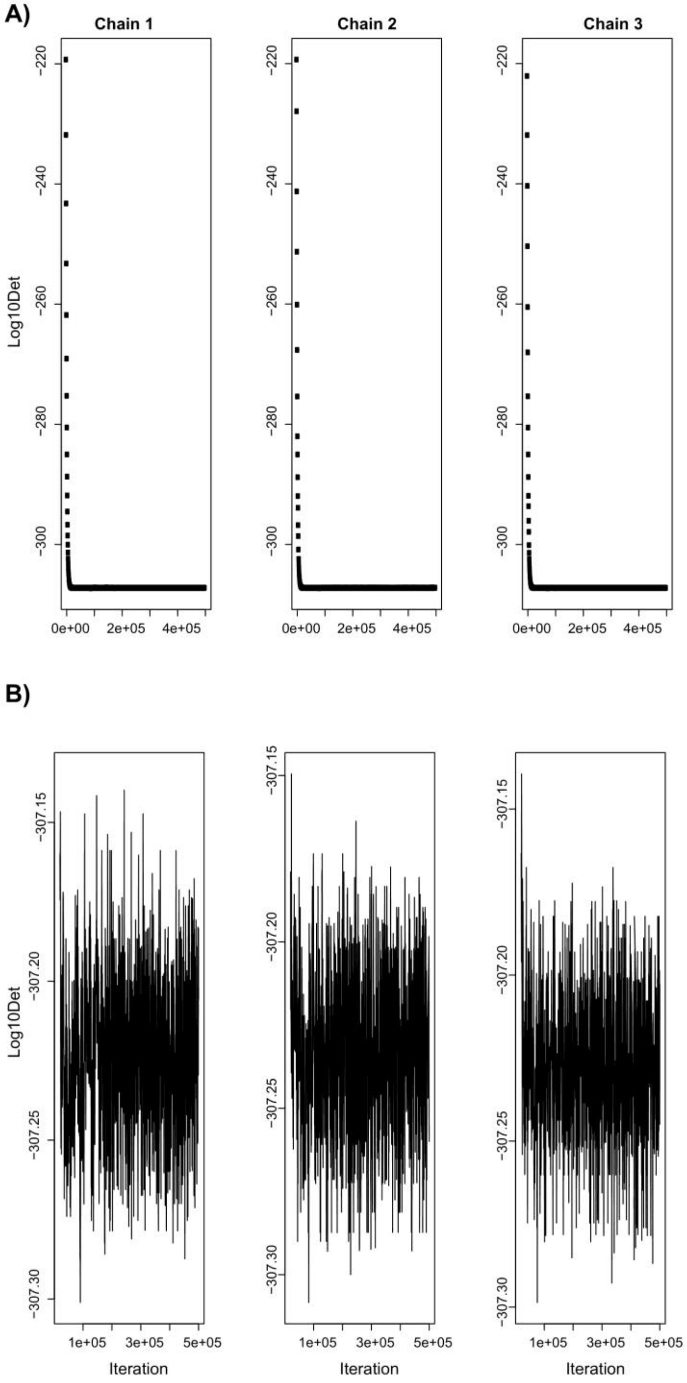

Supplementary figure 5: Geographical representation of Bayenv analyses for a few environmental gradients.

Change in mean minor allele frequencies of SNPs at each of the 98 hybrid populations that were identified as being associated with FFP, Tmin_wt, RH_sp & bFFP. The background colour palette represents a raster map of the respective environmental gradients (vertical colour bar). Each population is represented by a coloured circle representing the mean minor allele frequency for the associated SNPs as identified through Bayenv (horizontal colour bar).

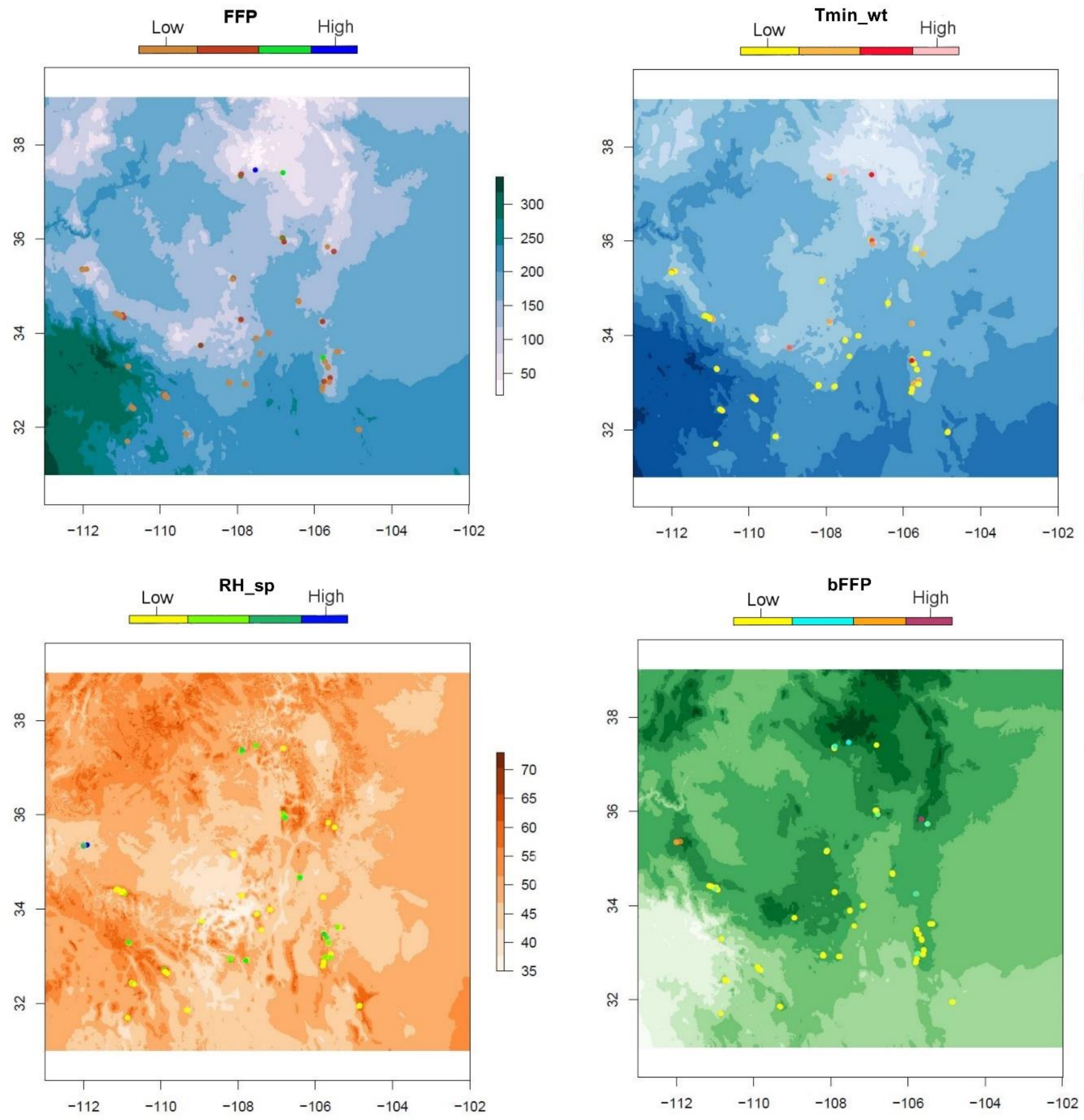

Supplementary figure 6: Scatterplots of change in population allele frequency along environmental gradients.

i-iv: Change in population allele frequency of outlier SNPs identified from Bayenv2 as a function of their respective environmental gradients. **(v:viii)** Change in population allele frequency of putatively neutral SNPs as a function of environmental gradients.

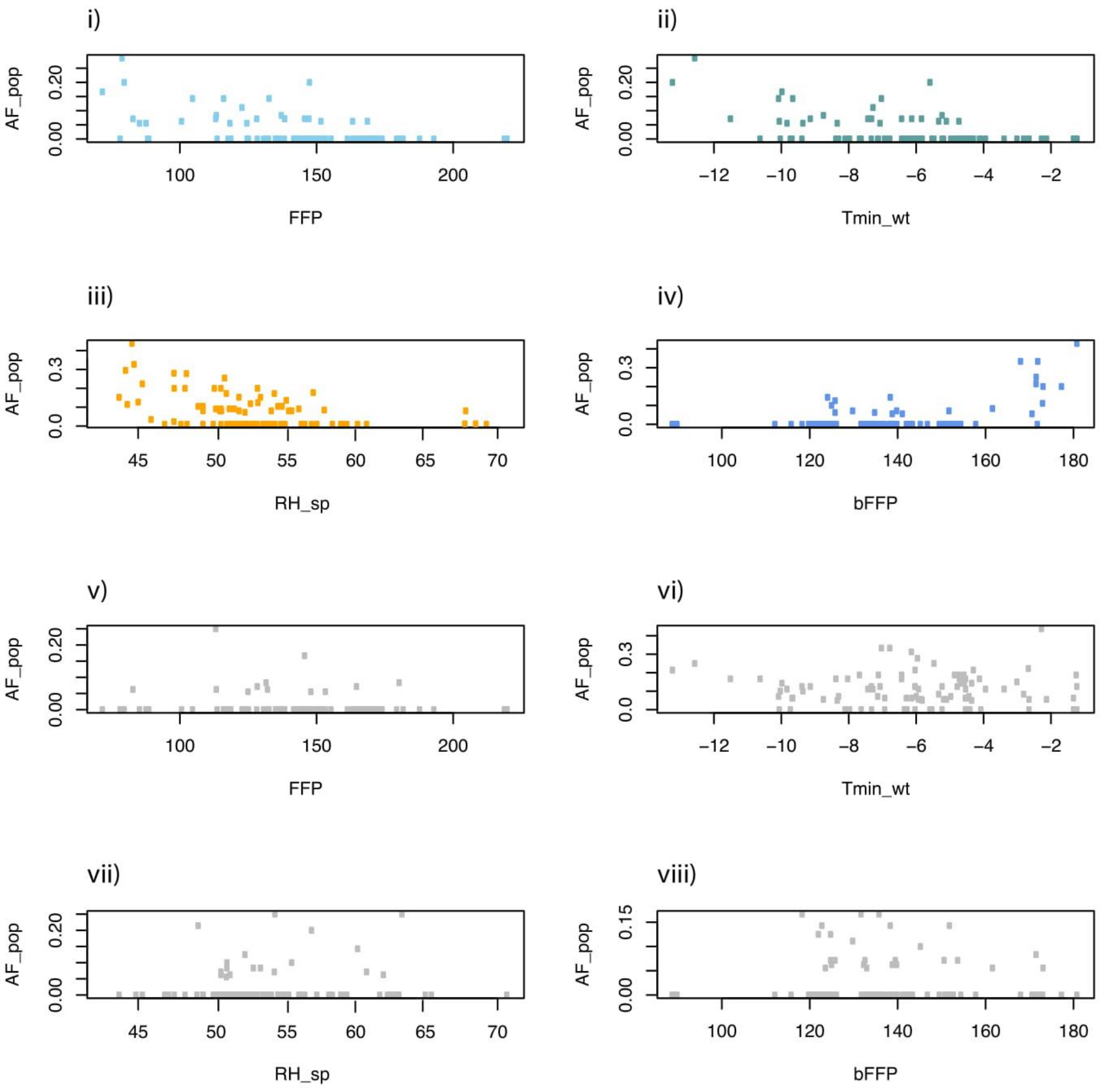

Supplement: Supplementary file 2 — Supplemental materials [file 42003_2020_1632_MOESM2_ESM.pdf]
