# Supplementary material for: Adaptive evolution in a conifer hybrid zone is driven by a mosaic of recently introgressed and background genetic variants
Source: Commun Biol. 2021 Feb 5;4:160. doi: 10.1038/s42003-020-01632-7 (PMC7864969; doi:10.1038/s42003-020-01632-7)
Supplement: Supplementary file 3 — Description of Supplementary Files [file 42003_2020_1632_MOESM3_ESM.pdf]

Description of Additional Supplementary Files

Supplementary Data 1: Explanation of all environmental gradients used in the present study and their mean value across the sampled range of pure *Pinus strobiformis* and *P. flexilis*. Seasons including winter, spring, summer and autumn encompass the following months: Winter = Jan, Feb & Dec; Spring = March to May; Summer = June to August; Autumn = September to November.

Supplementary Data 2: Summary statistics for Bayenv outlier SNPs associated with each of the 88 environmental gradients. Values in bold indicate significance at $p < 0.05$.
BF: median Bayes factor values across 3 replicate chains in Bayenv.
Noutliers: number of bayenv outliers as determined through the intersection of BF and |rho| detailed in the methods section of the main text.

Supplementary Data 3: Observed correlations for each pair of environmental gradients and the number of Bayenv outlier SNPs shared between them.
Overlap: Number of overlapping bayenv outlier SNPs, *perOverlap*: Percent overlapping

Supplementary Data 4: Putative functional annotation of freeze related dDocent contigs using *Pinus lambertiana* v.1.0 genome as the reference.
Column names are standard output from blastfmt 6.
PILAloc: name of the contig in *P. lambertiana* v.1.0 genome, *dDocentloc*: name of the contig in the denovo assembled genome used in this study.

Supplementary Data 5: Proportion of times sets of three, four, five and six Bayenv outliers were represented in the same OCs.

Supplementary Data 6: R^2 estimates and the corresponding p-values from the multiple matrix regression for D_{IS} and D_{ST} across the 88 environmental gradients. For gradients with 10 or fewer unique response values, only the R^2 is reported.

Supplementary Data 7: Fold enrichment (FE) estimates as obtained from genomic cline analyses for all 88 environmental gradients.

Supplementary Data 8: Loadings of all 88 environmental gradients on the top seven PC axes that were used as the environmental matrix in RDA.
