# Supplementary material for: Adaptive evolution in a conifer hybrid zone is driven by a mosaic of recently introgressed and background genetic variants
Source: Commun Biol. 2021 Feb 5;4:160. doi: 10.1038/s42003-020-01632-7 (PMC7864969; doi:10.1038/s42003-020-01632-7)
Supplement: Supplementary file 12 — Reporting Summary [file 42003_2020_1632_MOESM12_ESM.pdf]

nature research

Corresponding author(s): Mitra Menon

Last updated by author(s): Oct 22, 2020

Reporting Summary

Nature Research wishes to improve the reproducibility of the work that we publish. This form provides structure for consistency and transparency in reporting. For further information on Nature Research policies, see our Editorial Policies and the Editorial Policy Checklist.

Statistics

For all statistical analyses, confirm that the following items are present in the figure legend, table legend, main text, or Methods section.

n/a	Confirmed	
☐	☒	The exact sample size (n) for each experimental group/condition, given as a discrete number and unit of measurement
☐	☒	A statement on whether measurements were taken from distinct samples or whether the same sample was measured repeatedly
☐	☒	The statistical test(s) used AND whether they are one- or two-sided<br>*Only common tests should be described solely by name; describe more complex techniques in the Methods section.*
☐	☒	A description of all covariates tested
☐	☒	A description of any assumptions or corrections, such as tests of normality and adjustment for multiple comparisons
☐	☒	A full description of the statistical parameters including central tendency (e.g. means) or other basic estimates (e.g. regression coefficient) AND variation (e.g. standard deviation) or associated estimates of uncertainty (e.g. confidence intervals)
☐	☒	For null hypothesis testing, the test statistic (e.g. F, t, r) with confidence intervals, effect sizes, degrees of freedom and P value noted<br>*Give P values as exact values whenever suitable.*
☐	☒	For Bayesian analysis, information on the choice of priors and Markov chain Monte Carlo settings
☐	☒	For hierarchical and complex designs, identification of the appropriate level for tests and full reporting of outcomes
☐	☒	Estimates of effect sizes (e.g. Cohen's d, Pearson's r), indicating how they were calculated

Our web collection on statistics for biologists contains articles on many of the points above.

Software and code

Policy information about availability of computer code

Data collection	Climate and soil data were procured from open source websites like ClimateWNA & SoilGrid. Genomic data was generated from DNA extracted from trees growing in natural stands. Full description of these is also provided in the main text under the methods section.
Data analysis	Initial processing of genomic data was conducted using dDocent and a combination of custom python scripts. Genotype environment association was conducted using Bayenv2. All other analyses were conducted in R using either custom code (see data availability) or publicly available libraries within R. The publicly available libraries used for data analyses are listed in the main text. All figures were generated in R v.3.3.2. Custom python and R codes used for data analysis have been made publicly available and is linked to under the data availability statement.

For manuscripts utilizing custom algorithms or software that are central to the research but not yet described in published literature, software must be made available to editors and reviewers. We strongly encourage code deposition in a community repository (e.g. GitHub). See the Nature Research guidelines for submitting code & software for further information.

Data

Policy information about availability of data

All manuscripts must include a data availability statement. This statement should provide the following information, where applicable:

- Accession codes, unique identifiers, or web links for publicly available datasets
- A list of figures that have associated raw data
- A description of any restrictions on data availability

The raw fastq files have been made publicly available via NCBI SRA at PRJNA670193. These will be accessible post final acceptance. Minor allele 012 coded SNP file, outputs from major analyses run as a part of this manuscript and values of environmental variables at each population are provided at Figshare: 10.6084/m9.figshare.c.5130104.

Field-specific reporting

Please select the one below that is the best fit for your research. If you are not sure, read the appropriate sections before making your selection.

☐ Life sciences ☐ Behavioural & social sciences ☒ Ecological, evolutionary & environmental sciences

For a reference copy of the document with all sections, see nature.com/documents/nr-reporting-summary-flat.pdf

Ecological, evolutionary & environmental sciences study design

All studies must disclose on these points even when the disclosure is negative.

Study description	Our study intensely samples the hybrid zone formed between Pinus strobiformis and Pinus flexilis to evaluate the relative contribution of recently introgressed and background genetic variants towards adaptive evolution. For the hybrid zone we utilize a gridded sampling scheme. We also sample pure parental individuals to evaluate signatures of introgression.
Research sample	We extracted genomic DNA from needles collected from mature trees. Overall we sample 132 populations with a minimum of 3 trees per population and a maximum of 10 trees.
Sampling strategy	We collected populations across all of its distribution with an average distance among populations of ~ 50 km , except when the access to the localities was compromised by fires or narcotrafic. For each population we collected needles from an average of five trees.
Data collection	Data was collected and recorded based on the standard form that was used for the whole project. This included the date, locality, coordinates, slope, aspect, presence of alternative host and vegetation type. For each individual, coordinates and ID was recorded, ID was generated with three letters of the locality and the individual number. Trees were tagged with aluminum tags.
Timing and spatial scale	The samples were collected across Mexico and USA between 2014-2016.
Data exclusions	No data was excluded from this study.
Reproducibility	The coordinates of each tree that needles were collected from are recorded and can be made publicly available. The raw fastq files and all complete analyses pipeline has been made publicly available for reproducibility.
Randomization	We used a random number generator to multiplex individual trees within a plate. This layout was maintained throughout from DNA extraction to sequencing.
Blinding	Populations were allocated to either pure parentals or the hybrid zone based on a bayesian clustering approach that assigns each individual tree ancestral from P. strobiformis and P. flexilis. We declared populations as admixed if more than 80% of the trees within it contained admixed genomic ancestry.

Did the study involve field work? ☒ Yes ☐ No

Field work, collection and transport

Field conditions	Samples were collected from mature trees under dry weather conditions.
Location	Samples were collected from 132 locations ranging from Mexico to Wyoming. Information about the location of each individual tree will be publicly available post acceptance.
Access & import/export	For samples from Mexico: Secretaría de Medio Ambiente y Recursos Naturales (SEMARNAT), Mexico. Name of the issuing authority: SEMARNAT, date when it was issued: March 1st 2016, September 8th 20016, June 13th 2016.<br>USDA permits were used for collections from national forests in the USA when applicable.
Disturbance	We collected only few needles from each tree in a nondestructive manner.

Reporting for specific materials, systems and methods

We require information from authors about some types of materials, experimental systems and methods used in many studies. Here, indicate whether each material, system or method listed is relevant to your study. If you are not sure if a list item applies to your research, read the appropriate section before selecting a response.

Materials & experimental systems

n/a	Involved in the study
☒	☐ Antibodies
☒	☐ Eukaryotic cell lines
☒	☐ Palaeontology and archaeology
☒	☐ Animals and other organisms
☒	☐ Human research participants
☒	☐ Clinical data
☒	☐ Dual use research of concern

Methods

n/a	Involved in the study
☒	☐ ChIP-seq
☒	☐ Flow cytometry
☒	☐ MRI-based neuroimaging
